# Advancing the Clinical and Molecular Understanding of Cornelia de Lange Syndrome: A Multidisciplinary Pediatric Case Series and Review of the Literature

**DOI:** 10.3390/jcm13082423

**Published:** 2024-04-21

**Authors:** Karolina Gruca-Stryjak, Emilia Doda-Nowak, Julia Dzierla, Karolina Wróbel, Marta Szymankiewicz-Bręborowicz, Jan Mazela

**Affiliations:** 1 Department of Perinatology, Faculty of Medicine, University of Medical Sciences, 60-535 Poznan, Poland; 2 Department of Obstetrics and Gynecology, Polish Mother’s Memorial Hospital Research Institute, 93-338 Lodz, Poland; 3 Centers for Medical Genetics Diagnostyka GENESIS, 60-406 Poznan, Poland; 4 Faculty of Medicine, University of Medical Sciences, 61-701 Poznan, Polandjulia.dzierla@wp.pl (J.D.); 5 Department of Neonatology, Faculty of Medicine, University of Medical Sciences, 60-535 Poznan, Poland

**Keywords:** Cornelia de Lange syndrome, pediatric genetics, phenotypic variability, cohesinopathies, epigenetics

## Abstract

Cornelia de Lange syndrome (CdLS) is a complex genetic disorder with distinct facial features, growth limitations, and limb anomalies. Its broad clinical spectrum presents significant challenges in pediatric diagnosis and management. Due to cohesin complex mutations, the disorder’s variable presentation requires extensive research to refine care and improve outcomes. This article provides a case series review of pediatric CdLS patients alongside a comprehensive literature review, exploring clinical variability and the relationship between genotypic changes and phenotypic outcomes. It also discusses the evolution of diagnostic and therapeutic techniques, emphasizing innovations in genetic testing, including detecting mosaicism and novel genetic variations. The aim is to synthesize case studies with current research to advance our understanding of CdLS and the effectiveness of management strategies in pediatric healthcare. This work highlights the need for an integrated, evidence-based approach to diagnosis and treatment. It aims to fill existing research gaps and advocate for holistic care protocols and tailored treatment plans for CdLS patients, ultimately improving their quality of life.

## 1. Introduction

Cornelia de Lange syndrome (CdLS, OMIM 122470, 300590, 610759, 300882, 614701, 620568) is an essential model of pediatric congenital anomalies that poses multidimensional challenges to clinicians and researchers. This genetic disorder, characterized by distinctive facial features, growth retardations, and limb defects, is attracting attention because of its clinical heterogeneity and underlying genetic complexity. Impairments can affect multiple systems, including the cardiac, gastrointestinal, craniofacial, genitourinary, musculoskeletal, and central nervous systems, with developmental abilities ranging from profound intellectual disability to average intelligence with learning difficulties [1,2].

CdLS is estimated to occur in 1 in 10,000 to 30,000 live births, making it a significant concern in pediatric healthcare. The spectrum of the syndrome, from mild to severe forms, and the variety of possible presentations require astute clinical acumen for diagnosis [1,2]. Particularly challenging are the milder phenotypes, which can result from mosaic genetic patterns and require advanced genetic analysis to confirm the diagnosis.

However, the diagnosis can be made clinically (Table 1).

The diagnostic scale quantifies major (2 points) and minor (1 point) clinical features to facilitate a structured approach to diagnosis. Major features contributing to this scale include synophrys and thick eyebrows; an elongated, smooth fossa; a short, convex nose; a thin upper lip and lower corners of the mouth; oligodactyly or adactyly; and congenital diaphragmatic hernia [1]. Additional considerations include delayed intellectual development, intrauterine growth retardation, microcephaly, small hands and feet, a short fifth toe, and hirsutism. A cumulative score of 11 or more supports a diagnosis of classic CdLS, whereas a score of 9 to 10 indicates a non-classical variant. Scores in the intermediate range require molecular testing. If the patient scores less than four, there is no indication for molecular testing for CdLS [1].

Cohesin is a protein complex critical in gene expression and genome organization. It is involved in sister chromatid cohesion, DNA repair, transcriptional regulation, and genome stability. When mutations occur in cohesin or cohesin-regulatory genes, as in CdLS, they can disrupt the normal function of the complex and lead to dysregulation of gene expression, increased levels of oxidative stress, and genome instability. This can have wide-ranging effects on cellular processes and contribute to the developmental abnormalities observed in individuals with CdLS [3,4,5]. Pathogenic variants most likely cause CdLS in one of the cohesin complex genes, including *NIPBL (NIPBL cohesin loading factor-OMIM:608667)*, which accounts for about 50–60% of cases; *SMC1A (structural maintenance of chromosomes protein 1A-OMIM:300040)* and *HDAC8 (histone deacetylase 8-OMIM:300269)*, linked to the X chromosome; and *SMC3 (structural maintenance of chromosomes 3-OMIM:606062)* and *RAD21 (RAD21 cohesin complex component-OMIM:606462).* They can be spontaneous, autosomal dominant, or X-recessive [2,6]. Some of the newly identified genes that contribute to the understanding of CdLS include *ANKRD11 (ankyrin repeat domain-containing protein 11-OMIM:611192*), *EP300 (interacting inhibitor of differentiation 1-OMIM: 605894*), *AFF4 (alf transcription elongation factor 4-OMIM:604417*), *TAF1 (rna polymerase ii*, *tata box-binding protein-OMIM: 313650)*, *ARCN1 (coatomer protein complex-OMIM: 600820*), *ARID1B* (*at-rich interaction domain-containing protein 1b-OMIM: 614556)*, *ASXL2 (asxl transcriptional regulator 2-OMIM: 612991),* and *BRD4 (bromodomain-containing protein 4-OMIM:608749)* [3,7]. These additional genes are critical regulators of developmental transcriptional control, leading to the conditions they cause being disorders of transcriptional regulation (DTRs). Variants in these genes can result in a CdLS-like phenotype, expanding the genetic landscape of the syndrome [8]. In CdLS, the severity of the disease can vary even with the same genetic variants, suggesting that other modifying factors may play a role in influencing the outcome.

This article aims to provide a methodological review of pediatric case series and a comprehensive literature review on CdLS.

This article intends to serve as a resource for practicing clinicians and pediatricians by highlighting the genetic syndrome Cornelia de Lange syndrome. CdLS offers a unique opportunity to formulate a clinical diagnosis without molecular confirmation, especially in contexts where access to genetic services may be limited. Early diagnosis is essential for understanding the observed disorder’s pathophysiological aspects, which is crucial for initiating a multidisciplinary approach to care. Early diagnosis allows for tailoring therapeutic interventions and developmental support, paving the way for better patient care.

We are reviewing the extensive literature to synthesize findings from genetic studies, clinical reports, and therapeutic trials. Such synthesis is essential to identify the current state of knowledge and practice and to illuminate the way forward for pediatric CdLS care.

By consolidating this knowledge, we aim to develop clinical methodologies and stimulate further research, thus enriching pediatric genetic care.

This will lead to a more holistic understanding of the syndrome.

## 2. Case Series

This case series presents an insightful examination of four different cases of Cornelia de Lange syndrome managed at the Department of Neonatology, Gynaecology, and Obstetrics Hospital, Medical University of Poznan, Poland, from 2018 to 2023. The focus is on the clinical management and progression of the condition, following the journey from prenatal through labor and various stages of hospital care. Our narrative is dedicated to the critical decisions, therapeutic interventions, and follow-up approaches that embody the essence of CdLS management. Together, these four cases provide a comprehensive overview of the clinical course of the syndrome, highlighting the actionable aspects of pediatric care that are critical to improving outcomes for affected patients.

Case 1:

A baby girl weighing 1880 g (fetal growth restriction—FGR 1 point) was delivered by cesarean section due to life-threatening symptoms (deep late deceleration in cardiotocography monitoring) at 37 weeks gestation. The patient did not have antenatal testing. The patient did not have regular obstetric monitoring during pregnancy. An ultrasound scan performed two days before delivery showed intrauterine hypotrophy and a reduction of defects of the upper limbs in the fetus. The postnatal examination revealed hypotrophy (1 point), microcephaly (1 point), and numerous facial dysmorphic features, including a high forehead, synophrys (2 points), hypertelorism, narrow eyelid fissures, a short nose with an upturned tip (2 points), narrow red lips (2 points), an elongated philtrum (2 points), problems opening the mouth, short, contracted fingers, and small hands and feet (1 point). The baby had a small lower jaw and a shortened tongue frenulum, which prevented weight gain. She had a percutaneous endoscopic gastrostomy–jejunostomy (PEG-PEJ) to help with feeding. However, gastroesophageal reflux (GERD) continued to interfere with weight gain. The baby also required a tracheostomy due to tracheomalacia. Respiratory support was needed, and weaning from the ventilator was intolerable. In addition, imaging studies revealed a ventricular septal defect (VSD), a horseshoe kidney, and multiple renal pelvicoceles. Microarray-based comparative genomic hybridization (aCGH) analysis showed the presence of an interstitial deletion in the 2q13 region, including the NPHP1 gene, but this was unrelated to the patient’s phenotype. The baby was diagnosed with classic CdLS based on clinical features typical of CdLS, with a score of 12 points. After completing her initial hospitalization in the neonatal unit, the infant diagnosed with Cornelia de Lange syndrome was transferred to a specialist pediatric care and treatment facility. Unfortunately, there is no updated information on the patient’s progress following the transfer.

**Figure 1:**
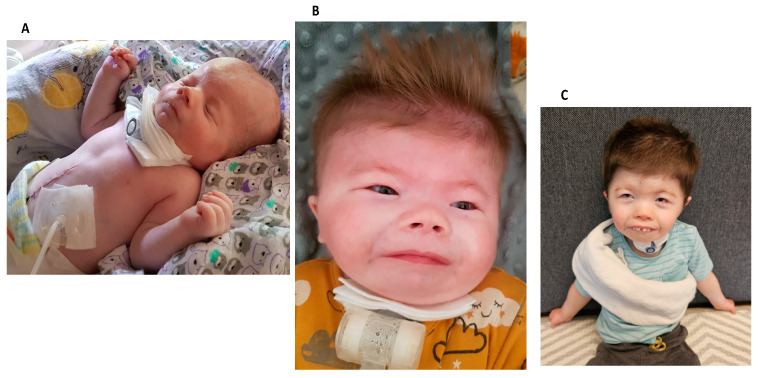
Clinical features of patients with Cornelia de Lange syndrome: Case 2: (**A**) patient on 25th day of life; (**B**) patient at 2 months of age; (**C**) patient at 3 years of age.

Case 2:

A male neonate weighing 1820 g (FGR = 1 point) was born spontaneously at 40 weeks gestation. The neonate scored 7 points at 1 min on the Apgar scale, and the umbilical cord blood pH was 7.15. Prenatal aCGH was performed because of FGR and suspected renal and brain developmental defects. The result was within the resolution limits of the test (100 kb). Postnatal examination revealed numerous dysmorphic features: microcephaly (1 point), square face, high and broad forehead, synophrys (2 points), coloboma oculi of the left eye, asymmetric and dysplastic auricles, flat and wide nasal bridge (2 points), bilateral choanal atresia, trismus, micrognathia, hypertelorism, short fifth fingers (1 point), syndactyly of the second and third toes (2 points), widely spaced nipples, and contractures of the limbs. Further imaging studies revealed the presence of VSD, diaphragmatic hernia (2 points), dysplasia of the right kidney, and cryptorchidism (Figure 1). The transfontanelle ultrasound (TFUI) showed abnormalities of brain structure: cortical–subcortical atrophy, numerous post-hemorrhagic cysts, slightly dilated lateral ventricles, hypoplasia of the corpus callosum, and enlargement of the choroid plexuses. Other impairments in the boy included growth retardation (1 point), arterial hypertension, congenital hypertonia, external auditory canal stenosis, mixed hearing loss, congenital pneumonia, GERD, and dysplastic acetabulum. The boy scored 11 points (Figure 1). The boy required mechanical ventilation and a gastrostomy for feeding. CHARGE syndrome was initially suspected. The child’s karyotype was evaluated to rule out the presence of a reciprocal break translocation in the 8q12.2 region related to the *CHD7* gene *(Chromodomain Helicase DNA Binding Protein 7-OMIM:608892)*. In the next step, whole-exome sequencing (WES) was performed and revealed a de novo pathogenic variant (c.883C > T variant) in the *HDAC8* gene.

Follow-up: At the age of four, the boy presented with psychomotor retardation and growth restriction. He sat but did not walk; he could hold objects correctly in his hands. He underwent mandibular distraction and gingival plasty (Figure 1). The boy has gastroesophageal reflux disease (GERD) and constipation. He enjoys uninterrupted sleep, indicating a stable nighttime pattern, but faces significant communication challenges due to a lack of language development, resulting in observable frustration and irritability during interactions. Despite these challenges, he is characterized by a generally cheerful disposition, which contrasts with his communication difficulties. Remarkably, he has not had any epileptic seizures to date.

Case 3:

A 1555 g female newborn (fifth pregnancy 5, birth 1, 36 weeks gestation) was delivered by cesarean section due to life-threatening fetal symptoms—decelerations in cardiotocography. The general condition of the newborn was described as average. The mother had gestational diabetes type 1. Prenatally, oligohydramnios, FGR (1 point), and upper limb defects (2 points) were noted. The newborn required non-invasive respiratory support. After birth, the physical examination revealed hypotrophy (1 point) and facial dysmorphic features: low hairline on forehead and nape of the neck, low forehead, synophrys (2 points), short nose (2 points), comprehensive, asymmetric, low-set auricles, thin upper lip (2 points), underdeveloped mandible and temporomandibular joint (TMJ), cleft hard palate, short, broad neck, marble skin, hirsutism (1 point), and widely spaced nipples. In addition, radiographic and orthopedic examination revealed deformed, shortened upper limbs, broad shoulder girdle, bilateral absence of ulna bones, flexion contracture of elbow and wrist joints, tiny feet (1 point), limited hip abduction, narrow hip girdle, and numerous other finger defects. The TFUI showed hypoplasia of the cerebellar vermis. In contrast, the TTE (transthoracic echocardiography) showed a patent foramen ovale, a VSD, and a bicuspid aortic valve with trace regurgitation. A tracheostomy was performed on the third day of life. The girl required mechanical ventilation until the 22nd day of life, followed by passive oxygen therapy. The girl also had hematological abnormalities such as coagulopathy and microcytic anemia (oral iron supplementation and erythropoietin treatment). The girl was fed partially parenterally and enterally; on the fourth day of life, stimulation of the sucking and swallowing reflexes was started, but without the expected effect. She was discharged from the hospital under the care of a nutrition clinic for intragastric feeding. At eight months of age, the above cardiological diagnoses were confirmed. Trace mitral regurgitation was also diagnosed, but cardiac dimensions and contractility were within the reference range. Weight gain in the first months of life was unsatisfactory (875 g at five months). She also had a global developmental delay (1 point) and hypotonia. The patient was diagnosed with classic CdLS based on clinical features typical of CdLS, scoring 11 points. aCGH analysis excluded chromosomal aberrations.

Follow-up: At the age of eight months, a percutaneous endoscopic gastrostomy (PEG) was placed. The girl suffered from GERD. The child’s psycho–physical development is delayed. The child’s height and weight remain below the third percentile. The child receives multidisciplinary medical care. The parents have not used genetic testing to confirm the disease’s molecular basis and establish genotype–phenotype associations.

**Figure 2:**
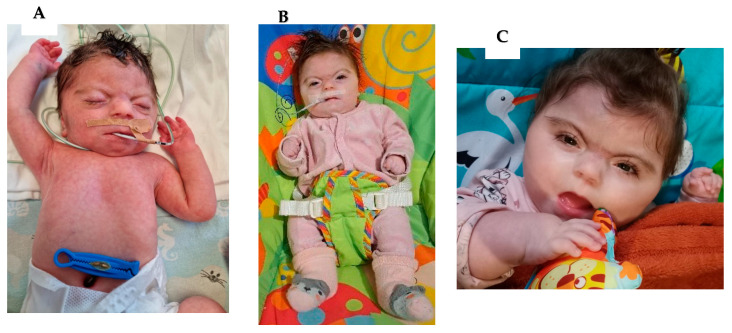
Clinical features of patients with Cornelia de Lange syndrome: Case 4: (**A**) patient at 5 days of life; (**B**) patient at 6 months of age; (**C**) patient at 13 months of age.

Case 4:

A female infant with intrauterine growth restriction, evidenced by a birth weight of 1600 g (1 point awarded), was delivered vaginally at 36 weeks gestation. The presence of meconium-stained amniotic fluid complicated the delivery. In addition, an abnormal umbilical cord with two vessels was diagnosed. Initially, the newborn required cardiopulmonary resuscitation, but by the time she was transferred to the neonatal unit, her circulatory and respiratory systems were functional. The mother had gestational diabetes type 1. Genetic amniocentesis was performed during pregnancy because of early FGR, but the result was in the normal range. Postnatal physical examination revealed numerous dysmorphic features (hirsutism (1 point), synophrys (2 points), long eyelashes, short nose with concave nasal ridge (2 points), thin upper lip vermilion (2 points), long philtrum (2 points), microcephaly (1 point), shortened forearms, broad hands with conical fingers, sunken chest, marble skin, hypertonic peripheral muscles, and weak signs of primitive neonatal reflexes). TTE showed a bicuspid aortic valve. The girl was fed an intragastric tube (foremilk and breastmilk supplement) from the fifth day of life due to a lack of sucking reflex (Figure 2). Classic CdLS was diagnosed based on clinical features typical of the syndrome (11 points in the clinical diagnostic classification). The girl had an intragastric feeding tube because of her intolerance to foremilk and pacifier feeding (presence of frequent regurgitation, abdominal pain, difficulty sucking, and lack of expected weight gain). After discharge from the neonatal unit, the infant, diagnosed with Cornelia de Lange syndrome, was placed under the supervision of specialist outpatient clinics, including cardiology, genetics, audiology, and nutrition (Figure 2).

Follow-up: At 13 months of life, the child was found to have significant deficits in body mass and growth, gastroesophageal reflux disease (GERD), and delayed psychomotor development. The parents are currently considering genetic testing for CDLs.

A summary of the clinical and diagnostic data for the four cases presented is given in Table 2.

## 3. Discussion

### 3.1. Clinical Aspects of Cornelia de Lange Syndrome

#### 3.1.1. Facial Gestalt, Malformations, Behavioral Problems

Cornelia de Lange syndrome is a paradigm in pediatric genetics that provides insight into the spectrum of congenital anomalies. The 2018 International Consensus provides a framework for classifying CdLS as either classical or non-classical, depending on clinical manifestations and genetic markers within chromatin-regulating genes, such as those constituting the cohesin complex [1].

Suspicion of CdLS is usually raised immediately after birth because of the characteristic phenotype [1,9,10]. A prominent philtrum with thin, sunken lips, narrow palpebral fissures, low-set ears, a depressed flat nasal bridge, anteverted nostrils, well-defined arched eyebrows with synophrys, long eyelashes, a cleft palate, microcephaly (small head), and micrognathia are some of the characteristic facial features associated with CDLS [11].

In our study, all cases showed the classic features of Cornelia de Lange syndrome, such as a distinctive facial gestalt, hirsutism, microcephaly, and growth restriction. These features are central to the clinical recognition of the syndrome, highlighting the importance of careful phenotypic assessment in diagnosis. The uniformity of these features across cases highlights the phenotypic consistency often observed in CDLS despite genetic variability.

CdLS is characterized by psychomotor retardation and intellectual disability. Learning disabilities range from mild to severe [12]. Children with CdLS often have musculoskeletal abnormalities in the upper extremities, ranging from small hands and feet to clinodactyly of the fifth finger to a missing forearm with digits. Although less common in the lower limbs, tiny feet and toe syndactyly may be seen in [13,14]. In discussing our article, it is worth noting that two of the cases described, specifically Case 1 and Case 3, presented a different spectrum of reduction defects of the upper limbs.

In contrast, cases with small hands and feet are characterized as 3 and 4. Clinodactyly was described in all cases. These phenotypic manifestations add to the spectrum of variability observed in the syndrome and highlight the heterogeneity of its clinical picture. CdLS is also associated with hip dysplasia, scoliosis, pectus excavatum, early development of bunions, and cervical spine abnormalities [15].

It should also be noted that, in addition to the clinical diagnostic criteria, other characteristic gastrointestinal symptoms or behavioral disturbances are common in patients with CdLS. Common gastrointestinal findings in Cornelia de Lange syndrome patients include gastroesophageal reflux, volvulus, rumination, chronic constipation, submucous cleft palate, sinusitis, and nasal polyps. Reflux may only present as difficulty swallowing, wheezing, vomiting, or aspirating food into the lungs. Silent reflux may be expected in CdLS. If there are no symptoms of reflux, looking for behavioral signs or signs of pain and discomfort may help diagnose [15,16]. All the newborns we described had feeding and gastro-reflux problems. Thanks to follow-up, we know that GERD is one of the main problems in Cases 2–4. Other findings may include Barrett’s esophagus, gastrointestinal problems such as the risk of volvulus, rumination, chronic constipation, and renal and urinary tract abnormalities [17,18].

One of CdLS’s most prominent behavioral features is self-injurious behavior (SIB), including head banging, hitting, or biting, which can be of considerable concern to carers. SIB has been reported in up to 70% of people with CdLS. It is considered a maladaptive coping mechanism and may be associated with frustration, difficulty communicating needs, sensory sensitivity, hyperactivity, impulsivity, and attention deficit [19]. Symptoms of attention deficit hyperactivity disorder (ADHD) have been observed in a significant proportion of people with CdLS [20]. In addition, individuals with CdLS may exhibit engagement (e.g., hand flapping), rigid adherence to routines, and repetitive vocalizations that interfere with adaptive functioning and social engagement [21,22]. Understanding and managing behavioral problems in CdLS requires a multidisciplinary approach, including behavioral interventions, occupational therapy, and pharmacological management as appropriate.

The parents of the Case 2 patient have reported behavioral problems, but these are related to hypersensitivity to stimuli, communication problems, and resulting frustration. In addition, the parents perceive the Case 2 patient as a child with a cheerful disposition; he does not display self-harming behavior or aggressive behavior toward others. The other cases described concern younger children and behavioral problems might appear later, so this aspect must be taken into account.

Congenital heart defects are present in approximately 25–30% of children with CdLS. Neurological disorders, such as epilepsy, are seen in up to 20% of cases of CdLS. People with CdLS may also have peripheral neuropathy, which can cause an abnormally high threshold for pain. Finally, structural abnormalities of the kidneys or urinary tract, such as vesicoureteral reflux, pelvic dilatation, and renal dysplasia, affect up to 40% of people with CdLS. There have also been reports of bicornuate or septate uteri, hypospadias, cryptorchidism, genital hypoplasia, and irregular menstruation [15]. In Case 2, defects were found in the internal organs. In the other cases, only facial gestalt and possibly limb defects existed despite the classic phenotype.

#### 3.1.2. Perioperative Care

Patients with Cornelia de Lange syndrome (CdLS) present unique challenges during anesthesia due to increased risk of perioperative stress, airway obstruction, and cardiovascular problems. Essential preoperative assessments, including a thorough physical examination of the airway and facial anatomy, are critical in identifying potential anesthetic complications. Cranial anomalies and micrognathia require careful planning and may benefit from advanced imaging studies such as CT or MRI to detect unrecognized airway abnormalities.

Anesthetic induction techniques should be carefully chosen to reduce stress and ensure airway stability. Inhalational anesthetics provide a gentle induction, avoiding the discomfort of needle-based methods. Conversely, intravenous induction with agents such as propofol or etomidate may be preferred for their advantages in airway management, seizure prevention, and minimization of post-induction agitation, provided they are administered with precise dose control. Percutaneous tracheostomy is a viable long-term strategy for ongoing airway management [23,24].

Postoperative care, particularly in the post-anesthesia care unit (PACU), must include vigilant monitoring for potential airway complications such as stridor or hypopnea to ensure patient safety and recovery. These considerations emphasize the need for tailored anesthetic protocols in patients with CdLS and highlight the importance of multidisciplinary collaboration in their care [25,26].

#### 3.1.3. Adolescent and Adult CdLS Problems

Diagnosing CdLS in adults with milder involvement can be challenging as it can resemble other conditions such as fetal alcohol spectrum disorder, Rubinstein–Taybi syndrome, and autism spectrum disorder. Recognizable adult facial features of CdLS include synophrys (joined eyebrows), narrow downward slanting palpebral fissures, a short nose with an anteverted nasal bridge, a prominent philtrum, thin downturned lips, and a square chin.

CdLS’s average adult growth parameters for weight, height, and head circumference are lower than standard growth curves. Most individuals were below the 5th percentile for growth in all parameters and had microcephaly. Some patients were also obese or had a low weight for their height [27,28].

Facial features in CdLS develop over time, becoming slightly coarser and longer. Typical facial features include synophrys, narrow palpebral fissures, a prominent philtrum, a short nose with an anteverted nasal bridge, jaw abnormalities, and downturned corners of the mouth. Gastrointestinal manifestations in CdLS include gastroesophageal reflux, esophagitis, gastritis, duodenitis, esophageal stricture, hiatal hernia, and gastrointestinal malformations. Other problems, such as constipation, chronic diarrhea, dysphagia, lactose intolerance, and milk protein allergy, are also reported [28,29].

The most common medical complication in CdLS is gastroesophageal reflux disease (GERD), which can manifest without apparent signs or symptoms. The study mentions a higher incidence of Barrett’s esophagus, a complication of GERD, in CdLS patients compared to the general population. This finding raises the possibility of silent reflux and the need for regular gastrointestinal evaluation in CdLS patients to address complications such as esophageal metaplasia and Barrett’s esophagus [30].

Janek et al. indicate that 65% of patients have sensorineural hearing loss, usually mild to moderate. Dental problems include crowding, delayed eruptions, missing teeth, dental caries, and bruxism [31].

A total of 41% of patients had ptosis (drooping of the eyelid), most of which was unilateral and did not require surgery [32]. A total of 71% of patients had blepharitis, an inflammation of the eyelids [33].

Scoliosis was documented in 39% of patients, and thoracic kyphosis in 7% [29].

A total of 80% of patients had hypertrichosis (excessive hair growth), and 61% had cutis marmarata (a marbled appearance of the skin).

The delayed onset of puberty was seen in both males and females.

More than two-thirds of the families reported sleep problems. These included frequent waking during the night, minimal sleep requirements (only 2–4 h per night), and the ability to go without sleep for extended periods (a maximum of three days) [34].

Neurodevelopmental manifestations included high pain tolerance, seizures, and varying degrees of intellectual disability [12].

Behavioral manifestations included self-injury, aggression, attention deficit disorder, wandering behavior, anxiety, depression, obsessive–compulsive tendencies, autistic-like behavior, and substance abuse problems [19,21,35].

This emphasizes the need for consistent medical care and stresses the role of a balanced diet and regular exercise. Transitioning from pediatric care to internal medicine or family practice is also crucial. Regular check-ups by ophthalmologists and dentists are recommended, especially to monitor conditions such as high myopia. Proactive management and continuous gastrointestinal (GI) surveillance are recommended for gastroesophageal reflux disease. An upper GI series is suggested to rule out malrotation, and any evidence of bowel obstruction or volvulus warrants immediate emergency department intervention, possibly requiring surgery. Hormone therapy is recommended for both contraception and menstrual regulation [18,29].

In Poland, these patients have been cared for by a multidisciplinary team that has worked for many years at the Medical University of Gdansk [36].

“The Cornelia de Lange Syndrome World/Poland Association” facilitates the meeting of patients and their relatives [37,38].

#### 3.1.4. Carer and Family of a CDLS Patient

Carers of people with Cornelia de Lange syndrome face significant challenges, mainly due to the complex medical profile of the syndrome. They often have to deal with the intensive management of multiple health problems, including gastrointestinal complications, cardiac anomalies, and developmental delays that require specialized medical care [39]. Behavioral difficulties, such as self-injury and aggression, require constant vigilance and specialized behavioral management strategies. The need for ongoing care and navigation of multiple medical systems can lead to carer fatigue [40]. In addition, ensuring effective communication with healthcare providers and advocating for the individual’s medical needs are constant challenges, adding to these carers’ emotional and logistical demands [36].

### 3.2. Deciphering the Genetic Puzzle: Advancements in Understanding Cornelia de Lange Syndrome

#### 3.2.1. Cohesin Complex

The molecular basis of CdLS is based on mutations in genes of the cohesin complex, which is responsible for chromatin stabilization, chromosome segregation, regulation of gene expression, and DNA repair [41,42,43].

Cohesin helps regulate the three-dimensional organization of the genome by bringing distant genomic regions together, which allows for enhancer–promoter interactions and facilitates gene transcription. Cohesin also plays a role in chromatin looping, insulating genome regions, and preventing promiscuous interactions between regulatory elements [44]. Moreover, cohesin regulates ribosomal RNA synthesis in nucleoli, promotes protein translation, and may affect chromosome arrangement throughout nucleoli [45].

The mitotic cohesin apparatus consists of four integral subunits: structural maintenance of chromosomes 1A (*SMC1A)* and structural maintenance of chromosomes 3 (*SMC3)*, which together form a heterodimeric configuration; *RAD21*, a cohesin complex component active during mitosis (or its meiotic counterpart, REC8: meiotic recombination protein); and cohesin subunit SA (STAG), with STAG1/STAG2 functioning in mitosis and STAG3 in meiosis. These subunits assemble to form the cohesin ring. The cohesin complex is loaded onto chromatids during the G1 phase of the mitotic or meiotic cell cycle by the cooperative function of the cohesin loading factor (*NIPBL)* and the sister chromatid cohesion factor *MAU2 (OMIM:614560)*. For successful cohesin loading, *NIPBL* must first bind to *MAU2. MAU2* interacts with the cohesin ring in the closed state, and its ATPase heads are engaged with ATP. During the S phase, cohesion establishment is intimately linked to DNA replication and requires that both sister chromatids are entrapped within the cohesin ring. This ring must also stay closed to prevent premature release of sister chromatids. Once the alignment of sister chromatids is complete, the anaphase promoter complex (APC) ubiquitinates securin, thereby releasing separase that will cleave RAD21 and dissociate the rest of the cohesin complex from the centromeres to allow segregation of sister chromatids. At the end of mitosis, ESCO2-induced acetylation of SMC3 during the S phase is reversed by histone deacetylase 8 (*HDAC8*), and SMCs are recycled and re-loaded onto chromatin [46]. Understanding these dysregulated biological pathways is crucial in comprehending CdLS as they shed light on the molecular mechanisms underlying the syndrome [47]. Seven genes are associated with CdLS (*NIPBL*, *SMC1A*, *SMC3)*, *BRD4*, *HDAC8*, *RAD21*, and *ANKRD11*), and several genes on different chromosomes contribute to its complex development [7,48,49]. They can be divided into two primary classifications: those encoding regulatory proteins of the cohesin complex, such as *NIPBL* and *HDAC8*, and those encoding structural components of the cohesin complex, such as *SMC1A*, *SMC3*, and *RAD21*. The latter trio, *SMC1A*, *SMC3*, and *RAD21*, are integral components of the cohesin complex and are responsible for its core architectural framework.

The Nipped-B-like (*NIPBL*) protein on chromosome 5p13.2 accounts for approximately 60% of cases, with the remaining genes accounting for 10%. Gene encoding protein is essential for recruiting the cohesin complex to chromatin. Pathogenic variants in NIPBL lead to reduced levels of functional NIPBL, resulting in haploinsufficiency and typically causing a severe CdLS phenotype known as the “classic” form.

One of the critical functions of *SMC1A* and *SMC3* is to regulate gene transcription and genome organization, ensuring correct chromosome segregation in mitosis and meiosis. They are also involved in the DNA damage repair (DDR) pathway. *The kinases ATM and ATR phosphorylate*, *SMC1A*, *and SMC3* are critical in the DNA damage response [50]. *SMC1A* and *SMC3* are not just components but crucial elements of the recombination protein complex (RC-1), which is involved in DNA repair by recombination. This underlines their role in preserving genome stability and integrity. Concerning CdLS, it is noteworthy that individuals with pathogenic variants in *SMC1A* and *SMC3* tend to exhibit milder phenotypes than those with *NIPBL* variants [47].

*HDAC8* acts as a critical cohesin regulator by deacetylating the *SMC3* protein. This modulates the dissociation of cohesin from the chromatin structure, a crucial step in cell cycle progression and the maintenance of genomic stability [13].

*BRD4* and *ANKRD11* have recently been added to a list of genes causing CdLS [51]. *BRD4* encodes chromatin-associated proteins which cooperate with *NIPBL* in transcriptional regulation. Variants in *BRD4* have been found in individuals with CdLS [48]. *ANKRD11*, on the other hand, is involved in regulating gene expression through chromatin remodeling, and variants in this gene have been reported in individuals with non-classical phenotypes [52].

Mutations affecting chromatin modifications due to cohesin abnormalities in CdLS lead to global transcription disturbances by causing perturbations in gene expression [3,53]. Studies have shown that in CdLS, cohesin mutations result in reduced cohesin binding to differentially expressed genes, while the binding is slightly reduced in non-differentially expressed genes. This leads to stable cohesin–DNA binding and impairs the recruitment of RNA polymerase II at the promoter regions of dysregulated genes. As a result, gene transcription is dysregulated in CdLS, with up to 1000 genes found to be dysregulated, suggesting that the phenotypic consequences arise from collective perturbations in gene expression [54].

The remaining thirty percent of CdLS patients are classified as idiopathic. Almost all cases of the condition are sporadic (de novo variants). However, an X-linked dominant pattern or autosomal dominant inheritance has been reported [55].

#### 3.2.2. Genotype–Phenotype Correlation

The complexity of CdLS far exceeds the scope of clinical diagnostic criteria, and knowledge of the location of specific disease-causing variants may offer opportunities to predict the future of a given patient [1,7,20]. Typical classical CdLS is a pathogenic variant in the *NIPBL* gene, and non-classical CdLS mutations are in the *SMC1A*, *RAD21*, and *ANKRD11* genes [20,56]. The genetic mechanisms of mutation in genes involved in CdLS can include various pathogenic variants, such as missense, truncating, or in-frame deletions, with the severity of the resulting phenotype influenced by additional modifying factors. Truncating variants accounted for most variants (46%), with 43% being deletions, 19% duplications, 2% insertions/deletions causing frameshifts, and 36% nonsense variants leading to premature protein truncation [7]. The clinical severity observed in Cornelia de Lange syndrome is significantly influenced by gene dosage effects, which are critical in determining the manifestation of the syndrome. There appears to be a gradation in the severity of the phenotype, with missense mutations typically resulting in milder forms of the disorder. In contrast, patients with nonsense mutations or large deletions have more severe manifestations. This grade supports the hypothesis that the *NIPBL* gene is sensitive to variations in gene dosage [4]. Patients with a pathogenic variant in the *NIPBL* gene have the most severe phenotypic features of classic CdLS [57]. This is particularly true for individuals carrying loss-of-function variants such as microdeletions, exon deletions, truncating, nonsense, splicing, and frameshift mutations. Such variants are uniquely associated with classic limb reduction anomalies. Conversely, patients with missense mutations typically have a more favorable outlook regarding clinical presentation and developmental outcomes [58]. Additionally, Nizon indicates that truncating mutations in exons 2–9 of *NIPBL* are associated with a reduced incidence of limb abnormalities and tend to have milder phenotypic effects than truncating mutations in exons 11–47 [59].

In Cases 1 and 3 described, limb reduction abnormalities and the classic phenotype suggest a high likelihood of Cornelia de Lange syndrome, possibly related to mutations in the NIPBL gene. This suggestion is consistent with the established genotype–phenotype correlations described above. Unfortunately, due to a lack of parental consent for genetic testing, we could not confirm the genetic basis in these cases, which limits our ability to provide tailored genetic counseling and anticipatory clinical care.

Patients carrying *SMC1A*, *SMC3*, and *RAD21* pathogenic variants share a mild to moderate phenotype characterized by craniofacial dysmorphism, impaired growth, and malrotations. *SMC1A* pathogenic variants can lead to two distinct phenotypes. Missense and frame-preserving del/dup variants result in a typical CdLS phenotype, affecting both males and females. On the other hand, truncating variants in *SMC1A* cause a severe neurocognitive seizure disorder known as “*SMC1A*-related neurocognitive disorder”. This disorder is characterized by epilepsy and significant neurocognitive impairment, resembling Rett syndrome (OMIM: 312750). Individuals with *SMC1A* truncating variants may be small and have microcephaly, but they do not exhibit the typical features of CdLS [56,60]. Cardiac defects were found in a small percentage of SMC1A (15%) and SMC3 (19%) cases, with a small percentage also having cleft palates (3–8%) [61,62]. *RAD21* variants result in similar features but milder cognitive involvement [49]. De Falco and colleagues documented novel clinical manifestations associated with a mutation in the *RAD21* gene. The index patient exhibited classic signs of Cornelia de Lange syndrome type 4, as well as previously unreported features such as cardiac anomalies, a cleft palate, and laryngomalacia [63].

In contrast, patients with a pathogenic variant in the *HDAC8* gene (5% of cases of CdLS) most commonly present with non-classical CdLS, but cases of patients fulfilling the criteria for the classical syndrome have been described. *HDAC8* gene variants lead to severe cognitive delay with specific facial features like delayed closure of the anterior fontanelle, hooded eyelids, widely spaced eyes, broad nose, and happy personality [64]. Male patients are more affected because the gene locus is on the X chromosome. The cognitive impairment observed is generally more severe than in patients with mutations in the *SMC1A*, *SMC3*, *BRD4*, and *RAD21* genes [65]. Causative variants in *HDAC8* range from CNVs (copy number variations) to SNVs (single nucleotide polymorphisms) [7]. In discussing Case 2, we note that the subject presented with a classic CdLS phenotype despite the pathogenic mutation in *HDAC8*. The child had renal anomalies and a diaphragmatic hernia, consistent with known *HDAC8*-associated clinical variations. Although the developmental delays are significant, the absence of epileptic seizures and the child’s remarkably cheerful disposition are consistent with the manifestations of the *HDAC8* variants previously reported in the literature. The boy presented with a diaphragmatic hernia, a rare abnormality in Cornelia de Lange syndrome (CdLS), suggesting a potential need for further investigation of its association with variants in the HDAC8 gene.

Constipation and GERD are more common in patients with variants in the *NIPBL* and *SMC1A* genes, visual impairment in *NIPBL*, *SMC1A*, and *HDAC8*, structural brain abnormalities in *NIPBL*, and sleep problems in *NIPBL* and *SMC1A* [56,66]. Dysmorphic features that meet the clinical diagnostic criteria for CdLS can also be seen in people with changes in the *BRD4*, *RAD21*, or *ANKRD11* genes, although they are not as severe or common [7,51]. Comprehensive molecular analysis in a cohort of 716 probands with CdLS revealed that causative variants in genes such as *AFF4*, *ANKRD11*, *ARCN1*, *ARID1B*, *ASXL2*, and others account for a significant proportion of etiologies beyond the traditional CdLS genes (25% of probands) [7]. These additional genes fall into categories such as causing syndromes with overlapping phenotypes, functioning in cohesin pathways, or potentially being novel CdLS genes.

Causative variants in additional genes, including *ANKRD11*, *EP300*, *AFF4*, *TAF1*, and *BRD4*, can cause CdLS-like phenotypes [47]. The *ANKRD11* gene, initially associated with KBG syndrome (OMIM: 148050), has also been linked to CdLS due to the remarkable phenotypic overlap between the two syndromes. Parenti et al. presented clinical and molecular details of 23 patients with *ANKRD11* variations and found that all but 2 were initially diagnosed with either CdLS or KBG syndrome. Only in five cases did the phenotype lead to a definitive diagnosis of KBG syndrome at later stages of development [67].

MAU2 is a gene that plays a crucial role in interacting with *NIPBL* to form the cohesin loader complex, known as kollerin. A pathogenic variant in *MAU2*, such as the p.(Gln310_Ala316del) mutation described by Parenti et al., can impair heterodimerization with NIPBL, affecting the stability of both proteins, leading to a phenotype resembling that of individuals with NIPBL mutations [68]. Specific symptoms reported in CdLS cases caused by *MAU2* variants include classic CdLS symptoms, severe mental, language, and motor disabilities, brain, hard, and kidney structural abnormalities, and growth retardation. It has been noted that the disease caused by *MAU2* mutation may vary in presentation, with some patients showing classic CdLS symptoms. In contrast, others may have different conditions or additional genetic abnormalities [69].

While the clinical diagnosis of Cornelia de Lange syndrome can be made based on characteristic dysmorphic features, molecular diagnosis is crucial for accurate genetic counseling and establishing genotype–phenotype correlations that directly impact the management and comprehensive care planning of affected individuals.

#### 3.2.3. Molecular Diagnostics

Molecular diagnosis is based on next-generation sequencing (NGS) of exomes (WES), targeted gene panels, or whole-genome sequencing (WGS) [1,7]. However, each has significant limitations. Sequencing analysis often detects variants of uncertain clinical significance, and their evaluation as causative variants requires an assessment of segregation within the family or the performance of functional studies. In addition, next-generation sequencing (NGS) technology cannot detect copy number variants. MLPA (multiplex ligation-dependent probe amplification) or aCGH (array comparative genomic hybridization) tests can also be performed to look for duplications or deletions in the *NIPBL* gene [7]. Difficulties in identifying disease-causing variants may arise from mosaicism or the coexistence of other genetic diseases. In the absence of mutations in CdLS-specific genes, mutations may be present in different cell lines (mosaicism) derived from tissues other than blood, such as fibroblasts.

There are still individuals with CdLS without an identifiable pathogenic variant, suggesting that additional mutational mechanisms beyond standard genetic screening may be involved, such as non-coding variants, deep intronic variants, and complex structural rearrangements.

Identifying these new candidate genes provides insight into the genetic heterogeneity of CdLS and related disorders. It enhances our understanding of these conditions’ molecular mechanisms and opens new avenues for research and potential therapeutic interventions.

#### 3.2.4. Mosaicism in CDLs

Mosaicism is a diagnostic and clinical dilemma in Cornelia de Lange syndrome (CdLS), illustrating the complex relationship of genetics in developmental disorders. Germline mosaicism, in which a subset of gametes carries mutations, but somatic cells are normal, has important implications for genetic counseling and the potential inheritance of genetic conditions in the absence of parental features. Somatic mosaicism, resulting from post-fertilization mutations, results in genetically diverse cell populations within an organism, profoundly affecting the expression of genetic diseases and often leading to less severe or atypical manifestations. Such variation can obscure precise diagnoses based on standard criteria for CdLS. In cases where CdLS is suspected, testing beyond blood samples may be required to diagnose accurately [70,71]. Next-generation sequencing techniques with high read depth ensure that sample collection is not limited to blood-derived DNA. Furthermore, Pozajevic et al. recommend using susceptible targeted sequencing methods, including pyrosequencing, to verify and quantify potential mosaic variants indicated by a limited number of sequencing reads [72].

Mosaicism, particularly in NIPBL and other cohesin complex genes, including SMC1A and HDAC8, underlies up to 20% of CdLS cases with characteristic features [73,74]. The unpredictable nature of mosaic mutations highlights the need for refined genetic counseling approaches for families affected by CdLS [75]. Unlike germline mutations with a more predictable inheritance pattern, mosaic mutations can occur spontaneously and unpredictably. This unpredictability requires a nuanced approach to counseling affected families.

#### 3.2.5. Epigenetics

Recent research suggests that epigenetic regulation may play a critical role in the pathogenesis of cohesinopathies. Cardiomyocyte cell lines with haploinsufficiency of *NIPBL* show alterations in numerous chromatin modifiers, ranging from histone modifiers to nucleosome remodeling and DNA-bending complexes. These findings suggest a direct or indirect involvement of *NIPBL* in the epigenetic regulatory mechanisms associated with Cornelia de Lange syndrome [76].

Epigenetic signatures, or episignatures, refer to distinctive patterns of epigenetic modifications, including DNA methylation, histone modification, and chromatin remodeling, without alterations to the underlying DNA sequence. Episignatures are characteristic of specific cell types, developmental stages, or disease states.

DNA methylation analysis is typically performed on DNA extracted from peripheral blood lymphocytes, using DNA methylation arrays or whole-genome bisulfite sequencing for greater accuracy down to a single nucleotide. The development of episignature profiling methods has become a tool for evaluating variants of unknown significance and distinguishing specific types of chromatinopathies (CPs) based on these unique DNA methylation patterns, which are specific to each CP case tested and differentiate between affected and unaffected individuals, as well as between disease-causing and non-disease-causing variants. Episignature profiling has also been used to assess mosaic variants, enabling disease monitoring when precision-targeted treatments are used to reverse the DNA methylation signature [77,78,79].

Over forty rare diseases have unique genome-wide DNA methylation patterns that are remarkably sensitive and specific to each disease. As a result, DNA methylation testing has been incorporated into the clinical diagnostic process for individuals with rare diseases. The complexity of these epigenetic marks and their potential reversibility position episignatures as a central focus for future biomedical research and therapeutic innovation [80,81,82].

Developed by Sadikovic and his team, EpiSign (a genome-wide DNA methylation assay) is the first commercially approved test for diagnosing genetic neurodevelopmental disorders using genomic DNA methylation analysis. Using machine learning algorithms, it assesses DNA methylation patterns in a patient’s peripheral blood and compares them to the comprehensive EpiSign Knowledge Database. This database includes reference samples covering many genetic disorders associated with developmental, intellectual, and other disabilities [79].

The EpiSign-CAN clinical trial is currently underway to validate the EpiSign test for diagnosing rare genetic diseases across Canada by gathering empirical evidence. This pivotal study will evaluate the efficacy, impact, and practicality of using EpiSign as an initial diagnostic tool and a secondary option following inconclusive results from comprehensive genetic and genomic analyses. In doing so, it aims to elucidate the diagnostic capacity of the test and its role in improving clinical pathways for patients with elusive genetic conditions [83] (https://episign.lhsc.on.ca/can.html (accessed on 10 March 2024)).

Kerkhof et al. studied 129 patients with cohesinopathies using Illumina Infinium EPIC arrays to generate DNA methylation data. Their study confirmed methylation profiles that matched sequencing results, providing critical insight into the diagnostic potential of the EpiSign test for Mendelian chromatinopathy disorders. The study’s authors also acknowledge the inherent limitations of the technique, which relies exclusively on recognized and established methylation profiles and is limited to the analysis of blood tissue types [84].

### 3.3. Prenatal Diagnosis

The diagnosis of CdLS after birth is predominantly clinical, based on the syndrome’s physical features. While a comparable antenatal approach is impossible, detailed fetal anatomy can be captured using high-resolution ultrasound. However, it is unrealistic to expect general sonographers to be familiar with such a rare condition [85].

Cardinal and suggestive features include distinct facial features such as thick, meeting eyebrows (synophrys), a short nose with a depressed nasal bridge, a long philtrum, a thin upper lip, and downturned mouth corners. Limb abnormalities such as oligodactyly, absence of bones, and congenital diaphragmatic hernia are also indicative, as are fetal growth restriction and microcephaly [86].

Advances in ultrasound technology, mainly 3D volumetric imaging, have improved the detection of these facial features. Even standard 2D ultrasound can detect abnormalities of the nose and philtrum. Limb defects, ranging from minor to complete absences, are detectable and often asymmetrical in CdLS. Congenital diaphragmatic hernia, classified as a cardinal feature, and growth restriction, including microcephaly, can be observed prenatally and are essential indicators of CdLS. In addition, a short fifth finger, often associated with trisomy 21, can also be seen in CdLS.

Other ultrasound findings in CdLS include increased nuchal translucency, long eyelashes, and facial micrognathia. Cardiac malformations, although not exclusive to CdLS, are also noted and are essential for prognosis and management. The text concludes by emphasizing the role of volumetric ultrasound in identifying the distinct phenotype of classic CdLS [86,87].

### 3.4. Differential Diagnosis

The diagnostic process for Cornelia de Lange syndrome involves a comprehensive evaluation to distinguish it from other conditions with similar clinical features.

Pathogenic variants associated with CdLS or CdLS-like phenotypes have been identified in genes involved in chromatin regulation such as *BRD4* and *AFF4* (CHOPS syndrome, OMIM 616368), *ANKRD11* (KBG syndrome, OMIM 148050), *EP300* (Rubinstein–Taybi syndrome, RSTS, OMIM 613684), *KMT2A* (Wiedemann–Steiner syndrome, WDSTS, OMIM 605130), and genes associated with Coffin–Siris syndrome (CSS), including *ARID1B* (OMIM 614556), *ARID1A* (OMIM 603024), *SMARCB1* (OMIM 601607), *SMARCA4* (OMIM 603254), *SMARCE1* (OMIM 603111), *ARID2* (OMIM 609539), *SOX11* (OMIM 600898), and *DPF2* (OMIM 601671). These disorders have notable clinical overlap with CdLS, particularly in terms of facial dysmorphism, intellectual disability, and growth and developmental delays, and are often considered in the differential diagnosis of CdLS. The clinical similarities between these syndromes may reflect underlying molecular interactions within shared pathways, with chromatin dysregulation being a common mechanistic step in the pathogenesis of these developmental disorders [48,52,88,89].

Smith–Lemli–Opitz syndrome (OMIM 270400) also has facial dysmorphism and intellectual disability. However, other features such as microcephaly, feeding difficulties, and limb abnormalities can distinguish it from CdLS. Although CdLS and Seckel syndrome (OMIM 210600) share some features, such as intellectual disability and growth retardation, Seckel syndrome is characterized by severe short stature, microcephaly, and distinctive facial abnormalities. Wolf–Hirschhorn syndrome (OMIM 194190) may present with facial dysmorphism similar to CdLS and intellectual disability. However, Wolf–Hirschhorn syndrome is distinguished by its specific pattern of facial features, such as the “Greek warrior helmet” appearance, seizures, and growth retardation [15,41].

Chung–Jansen syndrome (OMIM 617991) is a recently identified condition characterized by global developmental delay, intellectual disability, behavioral problems, obesity, and facial dysmorphism. Facial dysmorphism may include full eyebrows, synophrys, an upturned nose, large ears, tapered fingers, and bilateral clinodactyly of the fifth finger. Behavioral problems may consist of ADHD, autistic features, mood disorders, and anxiety disorders [90].

### 3.5. Future Perspectives

Recent research suggests promising therapeutic avenues for Cornelia de Lange syndrome, from targeting ribosome biogenesis to translation defects.

L-leucine supplementation, which has been shown to increase rRNA production and protein synthesis in zebrafish models of CdLS, activates the target of the rapamycin (TOR) pathway, which is critical for cell proliferation and ribosome biogenesis [45]. L-leucine partially rescues translational and developmental defects associated with zebrafish models of Cornelia de Lange syndrome [91]. In addition, lithium chloride (LiCl) treatment has demonstrated the potential to rescue neural developmental defects in zebrafish models and reduce cell death in CdLS patient fibroblasts through activation of the Wnt-β-catenin (WNT-wingless-type MMTV integration site family) and TOR pathways [92,93].

WNT signaling alterations in CdLS models have been associated with various CNS abnormalities. The canonical WNT pathway, essential for embryonic development, regulates cell proliferation, differentiation, migration, genetic stability, and apoptosis and maintains adult stem cells and pluripotent states. The malformations in CdLS may stem from disruptions in the developmental WNT/β catenin pathways [94].

Lithium modulates the WNT pathway and could benefit CdLS patients, although its prenatal use poses risks. Preclinical studies suggest that in utero exposure does not alter fetal development, but caution is advised. Postnatally, lithium might support neurodevelopment and is also considered for behavioral issues in conditions like fragile X syndrome. L-leucine, shown to rescue translation deficits in zebrafish CdLS models through mTOR pathway stimulation, presents another therapeutic avenue [95].

Recombinant human growth hormone (r-hGH) has also shown positive results in improving stature in CdLS patients. This suggests the efficacy of hormone therapy in addressing growth retardation [96].

Antioxidants targeting oxidative stress—linked to aging and neurodegeneration—have shown promise in extending the lifespan of CdLS cell cultures and alleviating symptoms in zebrafish models, making them a potential future treatment strategy [3,97].

Research indicates that N-acetyl-cysteine protects DNA from damage and inhibits cell death, particularly in cerebellar cells. A forthcoming randomized clinical trial is set to evaluate the effectiveness of N-acetyl-cysteine for treating neurodevelopmental disorders among patients with Cornelia de Lange syndrome (CdLS), with initiation anticipated this year as listed on the clinical trials database [98] (clinicaltrials.gov, NCT04381897, accessed on 7 March 2024).

There is currently an ongoing clinical trial evaluating the effectiveness of interventions in significantly reducing problem behaviors in children with CdLS. The trial uses a robust multidimensional approach to measure behavioral change, with success defined as an 80% reduction in disruptive behaviors such as aggression and tantrums [99] (clinicaltrials.gov, NCT05829668, accessed on 7 March 2024).

Initiatives to develop specific therapies for chromatinopathies or imprinting disorders are progressing, as evidenced by the initiation of clinical trials. These groundbreaking trials explore treatments that modulate epigenetic modifications using strategies such as small molecule inhibitors of histone deacetylases, antisense oligonucleotides, and interventions targeting DNA methylation and demethylation [100,101]. In addition, cutting-edge techniques such as CRISPR/Cas9 genome editing and CRISPR-driven RNA editing are being tested in [102].

Developing targeted therapies tailored to the underlying epigenetic dysregulation in diseases associated with chromatinopathies faces several key challenges and considerations. First, one challenge is identifying specific epigenetic markers, such as episignatures, unique to each disease subtype or subgroup. These episignatures serve as promising biomarkers for diagnosis and treatment, but ensuring their specificity and reliability in different patient populations is critical for the success of targeted therapies. Second, the complexity of epigenetic modifications and their interplay with genetic and environmental factors adds another layer of difficulty to developing targeted therapies for CPs. In addition, the dynamic nature of epigenetic changes over time and in response to various stimuli poses a challenge to maintaining the long-term efficacy of targeted therapies. Continuous monitoring and adjustment of treatment strategies based on the evolving epigenetic landscape in these patients is necessary to ensure optimal outcomes. Another consideration is the heterogeneity of CdLS, which encompasses a wide range of genetic disorders with diverse clinical presentations. Tailoring targeted therapies to the specific genetic mutations and epigenetic alterations in each CdLS subtype requires personalized approaches that consider individual variability in disease manifestation and response to treatment. Furthermore, the lack of FDA-approved targeted therapies highlights the importance of conducting rigorous clinical trials to evaluate the safety and efficacy of novel treatment modalities. In the future, collaborative efforts between researchers, clinicians, and regulatory agencies will be essential in translating promising therapeutic strategies into clinical practice that will benefit CP patients [103].

## 4. Conclusions

Cornelia de Lange syndrome (CdLS) is a rare genetic disorder with a wide range of clinical manifestations and genetic causes. Although considerable progress has been made in understanding CdLS, further research is needed to better define the genotype–phenotype correlations and develop targeted therapeutic interventions. The article also suggests investigating new CdLS genes that have not yet been identified and exploring hidden pathogenic variants in non-coding DNA fragments in known or new CdLS genes that may be missed by DNA-based screening.

The key findings from our case series highlight the clinical complexity of Cornelia de Lange syndrome (CdLS). Each case contributes to a deeper understanding of the syndrome’s spectrum, which includes limb malformations, unique facial features, and developmental delays. These findings advance our knowledge of CdLS, allowing for improved investigations that may pave the way for more nuanced management strategies and potential therapeutic avenues.

Research into the epigenetics of cohesinopathies holds great promise due to the availability and ongoing development of drugs that target specific epigenetic alterations. By identifying these novel candidate genes and understanding how they interact with cohesin-related pathways, we can gain valuable insights into the molecular mechanisms contributing to CdLS and related disorders, paving the way for improved diagnosis, management, and potentially targeted therapies. A collaborative and multidisciplinary approach involving health professionals, researchers, and carers is essential to improving the diagnosis, management, and quality of life of people with CdLS.

## Figures and Tables

**Table 1:** Criteria for clinical diagnosis of CdLS.

Clinical Features of CdLS [1]	
Cardinal Features (2 Points Each if Present)	Suggestive Features (1 Point Each if Present)
(1) synophrys (HP:0000664) and/or thick eyebrows (HP:0000574) (2) long (HP:0000343) and/or smooth philtrum (HP:0000319) (3) short nose (HP:0003196), concave nasal ridge (HP:0011120), and/or upturned nasal tip (HP:0000463) (4) thin upper lip vermilion (HP:0000219) and/or downturned corners of the mouth (HP:0002714) (5) hand oligodactyly (HP:0001180) and/or adactyly (HP:0009776) (6) congenital diaphragmatic hernia (HP:0000776)	(1) global developmental delay (HP:0001263) and/or intellectual disability (HP:0001249) (2) prenatal growth restriction (<2 SD) (HP:0001511) (3) postnatal growth retardation (HP:0008897) (4) microcephaly (HP:0000252) (5) small hands (HP:0200055) and/or feet (HP:0001773) (6) short fifth finger (HP:0009237) (7) hirsutism (HP:0001007)
≥11 points, at least three features are cardinal: classic CdLS	
9–10 points, at least two features are cardinal: non-classic CdLS	
4–8 points and at least one feature is cardinal: indication for molecular testing for CdLS	
<4 points: insufficient to indicate molecular testing for CdLS	

**Table 2:** Summary of the clinical and genetic findings of the clinical case series.

	Case 1	Case 2	Case 3	Case 4
Prenatal ultrasound findings	FGR	FGR	FGR	FGR
	upper limb defects	renal abnormality	upper limb defects	single umbilical artery
		brain defects	oligohydramnios	
Cardinal features	synophrys	synophrys	synophrys	synophrys
	short nose	flat and broad nasal bridge and short nose	short nose	short nose
	narrowed red lips	syndactyly of the 2nd and 3rd toes	thin upper lip	thin upper lip
	elongated philtrum	diaphragmatic hernia		long philtrum
	upper limb defects		upper limb defects	
Suggestive features	pre- and postnatal growth restriction	pre- and postnatal growth restriction	pre- and postnatal growth restriction	pre- and postnatal growth restriction
	microcephaly	microcephaly	microcephaly	microcephaly
	hirsutism	hirsutism	hirsutism	hirsutism,
	small hands and feets	short fifth finger	small feet	
	global developmental delay	global developmental delay	global developmental delay	global developmental delay
Cumulative score in clinical assesment	12 points	11 points	11 points	11 points
Genetics tests	aCGH (postnataly)—deletion 2q13	aCGH, karyotype (prenatally)—normal findings	aCGH (postnataly)—normal findings	aCGH (prenatally)—normal findings
		WES (postnataly)—pathogenic de novo variant in HDAC8 gene (c.883C>T)		

## Data Availability

The data presented in this study are available upon request from the corresponding author.

## Abbreviations

CdLS	Cornelia de Lange syndrome
FGR	fetal growth restriction
PEG-PEJ	percutaneous endoscopic gastrostomy–jejunostomy
GERD	gastroesophageal reflux
VSD	ventricular septal defect
aCGH	array comparative genomic hybridization
NGS	next-generation sequencing
MLPA	multiplex ligation-dependent probe amplification
WES	whole-exome sequencing
WGS	whole-genome sequencing
TFU	transfontanelle ultrasound
TMJ	temporomandibular joint
TTE	transthoracic echocardiography
PEG	percutaneous endoscopic gastrostomy
SIB	self-injurious behavior
ADHD	attention deficit hyperactivity disorder
PACU	post-anesthesia care unit
CNV	copy number variation
SNPs	single nucleotide polymorphisms
CPs	chromatinopathies

## References

[1] Kline A.D., Moss J.F., Selicorni A., Bisgaard A.-M., Deardorff M.A., Gillett P.M., Ishman S.L., Kerr L.M., Levin A.V., Mulder P.A. (2018). Diagnosis and management of Cornelia de Lange syndrome: First international consensus statement. Nat. Rev. Genet..

[2] de Lange C. Syndrome 1; CDLS1. https://www.omim.org/entry/122470.

[3] Sarogni P., Pallotta M.M., Musio A. (2020). Cornelia de Lange syndrome: From molecular diagnosis to therapeutic approach. J. Med. Genet..

[4] Selicorni A., Mariani M., Lettieri A., Massa V. (2021). Cornelia de Lange Syndrome: From a Disease to a Broader Spectrum. Genes..

[5] Bose T., Gerton J.L. (2010). Cohesinopathies, gene expression, and chromatin organization. J. Cell Biol..

[6] Mfarej M.G., Hyland C.A., Sanchez A.C., Falk M.M., Iovine M.K., Skibbens R.V. (2023). Cohesin: An emerging master regulator at the heart of cardiac development. Mol. Biol. Cell.

[7] Kaur M., Blair J., Devkota B., Fortunato S., Clark D., Lawrence A., Kim J., Do W., Semeo B., Katz O. (2023). Genomic analyses in Cornelia de Lange Syndrome and related diagnoses: Novel candidate genes, genotype–phenotype correlations and common mechanisms. Am. J. Med. Genet. Part A.

[8] Izumi K. (2016). Disorders of Transcriptional Regulation: An Emerging Category of Multiple Malformation Syndromes. Mol. Syndr..

[9] Rohatgi S., Clark D., Kline A.D., Jackson L.G., Pie J., Siu V., Ramos F.J., Krantz I.D., Deardorff M.A. (2010). Facial diagnosis of mild and variant CdLS: Insights from a dysmorphologist survey. Am. J. Med. Genet. Part A.

[10] Jackson L., Kline A.D., Barr M.A., Koch S. (1993). de Lange syndrome: A clinical review of 310 individuals. Am. J. Med. Genet..

[11] Collis L., Moss J., Jutley J., Cornish K., Oliver C. (2008). Facial expression of affect in children with Cornelia de Lange syndrome. J. Intellect. Disabil. Res..

[12] Cereda A., Mariani M., Rebora P., Sajeva A., Ajmone P.F., Gervasini C., Russo S., Kullmann G., Valsecchi G., Selicorni A. (2016). A new prognostic index of severity of intellectual disabilities in Cornelia de Lange syndrome. Am. J. Med. Genet. Part C Semin. Med. Genet..

[13] Dowsett L., Porras A.R., Kruszka P., Davis B., Hu T., Honey E., Badoe E., Thong M., Leon E., Girisha K.M. (2019). Cornelia de Lange syndrome in diverse populations. Am. J. Med. Genet. Part A.

[14] Mehta D., Vergano S.A.S., Deardorff M., Aggarwal S., Barot A., Johnson D.M., Miller N.F., Noon S.E., Kaur M., Jackson L. (2016). Characterization of limb differences in children with Cornelia de Lange Syndrome. Am. J. Med. Genet. Part C Semin. Med. Genet..

[15] Deardorff M.A., Noon S.E., Krantz I.D., Adam M.P., Feldman J., Mirzaa G.M., Pagon R.A., Wallace S.E., Bean L.J.H., Gripp K.W., Amemiya A. Cornelia de Lange Syndrome. GeneReviews® [Internet].

[16] Luzzani S., Macchini F., Valadè A., Milani D., Selicorni A. (2003). Gastroesophageal reflux and Cornelia de Lange syndrome: Typical and atypical symptoms. Am. J. Med. Genet. Part A.

[17] Gillis L.A., McCallum J., Kaur M., DeScipio C., Yaeger D., Mariani A., Kline A.D., Li H.-H., Devoto M., Jackson L.G. (2004). NIPBL Mutational Analysis in 120 Individuals with Cornelia de Lange Syndrome and Evaluation of Genotype-Phenotype Correlations. Am. J. Hum. Genet..

[18] Clermidi P., Abadie V., Campeotto F., Irtan S. (2015). Sigmoid Volvulus: An Underestimated Cause of Intestinal Obstruction in Cornelia de Lange Syndrome. J. Pediatr..

[19] Bell L., Oliver C., Wittkowski A., Moss J., Hare D. (2018). Attenuated behaviour in Cornelia de Lange and fragile X syndromes. J. Intellect. Disabil. Res..

[20] Moss J., Penhallow J., Ansari M., Barton S., Bourn D., FitzPatrick D.R., Goodship J., Hammond P., Roberts C., Welham A. (2017). Genotype–phenotype correlations in Cornelia de Lange syndrome: Behavioral characteristics and changes with age. Am. J. Med. Genet. Part A.

[21] Grados M.A., Alvi M.H., Srivastava S. (2017). Behavioral and psychiatric manifestations in Cornelia de Lange syndrome. Curr. Opin. Psychiatry.

[22] Moss J., Nelson L., Powis L., Waite J., Richards C., Oliver C. (2016). A Comparative Study of Sociability in Angelman, Cornelia de Lange, Fragile X, Down and Rubinstein Taybi Syndromes and Autism Spectrum Disorder. Am. J. Intellect. Dev. Disabil..

[23] Stevic M., Milojevic I., Bokun Z., Simic D. (2015). Unpredictable drug reaction in a child with Cornelia de Lange syndrome. Int. J. Clin. Pharm..

[24] Moretto A., Scaravilli V., Ciceri V., Bosatra M., Giannatelli F., Ateniese B., Mariani M., Cereda A., Sosio S., Zanella A. (2016). Sedation and general anesthesia for patients with Cornelia De Lange syndrome: A case series. Am. J. Med. Genet. Part C Semin. Med. Genet..

[25] https://www.orphananesthesia.eu/en/rare-diseases/published-guidelines/cornelia-de-lange-syndrome/285-cornelia-de-lange-syndrome/file.html.

[26] https://www.cdlsworld.org/xwiki/bin/download/cdlsPublications/medicalPassport/worldCarecard2018.pdf?rev=1.1.

[27] Nelson L., Moss J., Oliver C. (2014). A Longitudinal Follow-Up Study of Affect in Children and Adults With Cornelia de Lange Syndrome. Am. J. Intellect. Dev. Disabil..

[28] Wierzba J., Wierzba T., Mazurkiewicz-Bełdzińska M., Szyca R., Kozłowski J., Banach P., Potaż P., Limon J. (2010). Dorosły z Rzadkim Schorzeniem Genetycznym—Diagnostyka i Terapia Zespołu Cornelii de Lange. Forum Med. Rodz..

[29] Mariani M., Decimi V., Bettini L.R., Maitz S., Gervasini C., Masciadri M., Ajmone P., Kullman G., Dinelli M., Panceri R. (2016). Adolescents and adults affected by Cornelia de Lange syndrome: A report of 73 Italian patients. Am. J. Med. Genet. Part C Semin. Med. Genet..

[30] Pallotta M.M., Di Nardo M., Hennekam R.C.M., Kaiser F.J., Parenti I., Pié J., Ramos F.J., Kline A.D., Musio A. (2023). Cornelia de Lange syndrome and cancer: An open question. Am. J. Med. Genet. Part A.

[31] Janek K.C., Smith D.F., Kline A.D., Benke J.R., Chen M.-L., Kimball A., Ishman S.L. (2016). Improvement in hearing loss over time in Cornelia de Lange syndrome. Int. J. Pediatr. Otorhinolaryngol..

[32] Shi A., Levin A.V. (2019). Ophthalmologic findings in the Cornelia de Lange syndrome. Ophthalmic Genet..

[33] Avgitidou G., Cursiefen C., Heindl L. (2015). OOphthalmological manifestations of Cornelia de Lange syndrome: Case report and review of the literature. Ophthalmologe..

[34] Zambrelli E., Fossati C., Turner K., Taiana M., Vignoli A., Gervasini C., Russo S., Furia F., Masciadri M., Ajmone P. (2016). Sleep disorders in Cornelia de Lange syndrome. Am. J. Med. Genet. Part C Semin. Med. Genet..

[35] Kline A.D., Grados M., Sponseller P., Levy H.P., Blagowidow N., Schoedel C., Rampolla J., Clemens D.K., Krantz I., Kimball A. (2007). Natural history of aging in Cornelia de Lange syndrome. Am. J. Med. Genet. Part C Semin. Med. Genet..

[36] Wierzba J., Mazurkiewicz-Bełdzińska M., Jabłońska-Brudło J., Potaż P., Banach P. (2015). Challenges of caring for a patient with a rare disease--as demonstrated by Cornelia de Lange Syndrome. Dev. Period. Med..

[37] Cacioppo C.N., Conway L.J., Mehta D., Krantz I.D., Noon S.E. (2016). Attitudes about the use of internet support groups and the impact among parents of children with Cornelia de Lange syndrome. Am. J. Med. Genet. Part C Semin. Med. Genet..

[38] https://www.cdlsworld.org/xwiki/bin/view/Main/.

[39] January K., Conway L.J., Deardorff M., Harrington A., Krantz I.D., Loomes K., Pipan M., Noon S.E. (2016). Benefits and limitations of a multidisciplinary approach to individualized management of Cornelia de Lange syndrome and related diagnoses. Am. J. Med. Genet. Part C Semin. Med. Genet..

[40] Wulffaert J., van Berckelaer-Onnes I., Kroonenberg P., Scholte E., Bhuiyan Z., Hennekam R. (2009). Simultaneous analysis of the behavioural phenotype, physical factors, and parenting stress in people with Cornelia de Lange syndrome. J. Intellect. Disabil. Res..

[41] Avagliano L., Parenti I., Grazioli P., Di Fede E., Parodi C., Mariani M., Kaiser F.J., Selicorni A., Gervasini C., Massa V. (2020). Chromatinopathies: A focus on Cornelia de Lange syndrome. Clin. Genet..

[42] Meisenberg C., Pinder S.I., Hopkins S.R., Wooller S.K., Benstead-Hume G., Pearl F.M.G., Jeggo P.A., Downs J.A. (2019). Repression of Transcription at DNA Breaks Requires Cohesin throughout Interphase and Prevents Genome Instability. Mol. Cell.

[43] Litwin I., Pilarczyk E., Wysocki R. (2018). The Emerging Role of Cohesin in the DNA Damage Response. Genes.

[44] Schwarzer W., Abdennur N., Goloborodko A., Pekowska A., Fudenberg G., Loe-Mie Y., Fonseca N.A., Huber W., Haering C.H., Mirny L. (2017). Two independent modes of chromatin organization revealed by cohesin removal. Nature.

[45] Bose T., Lee K.K., Lu S., Xu B., Harris B., Slaughter B., Unruh J., Garrett A., McDowell W., Box A. (2012). Cohesin Proteins Promote Ribosomal RNA Production and Protein Translation in Yeast and Human Cells. PLoS Genet..

[46] Makrantoni V., Marston A.L. (2018). Cohesin and chromosome segregation. Curr. Biol..

[47] Piché J., Van Vliet P.P., Pucéat M., Andelfinger G. (2019). The expanding phenotypes of cohesinopathies: One ring to rule them all!. Cell Cycle.

[48] Olley G., Ansari M., Bengani H., Grimes G.R., Rhodes J., von Kriegsheim A., Blatnik A., Stewart F.J., Wakeling E., Carroll N. (2018). BRD4 interacts with NIPBL and BRD4 is mutated in a Cornelia de Lange–like syndrome. Nat. Genet..

[49] Deardorff M.A., Wilde J.J., Albrecht M., Dickinson E., Tennstedt S., Braunholz D., Mönnich M., Yan Y., Xu W., Gil-Rodríguez M.C. (2012). RAD21 Mutations Cause a Human Cohesinopathy. Am. J. Hum. Genet..

[50] Musio A. (2020). The multiple facets of the SMC1A gene. Gene.

[51] Shangguan H., Chen R. (2022). Phenotypes of Cornelia de Lange syndrome caused by non-cohesion genes: Novel variants and literature review. Front. Pediatr..

[52] Parenti I., Gervasini C., Pozojevic J., Graul-Neumann L., Azzollini J., Braunholz D., Watrin E., Wendt K.S., Cereda A., Cittaro D. (2016). Broadening of cohesinopathies: Exome sequencing identifies mutations in *ANKRD11* in two patients with Cornelia de Lange-overlapping phenotype. Clin. Genet..

[53] Yuan B., Pehlivan D., Karaca E., Patel N., Charng W.-L., Gambin T., Gonzaga-Jauregui C., Sutton V.R., Yesil G., Bozdogan S.T. (2015). Global transcriptional disturbances underlie Cornelia de Lange syndrome and related phenotypes. J. Clin. Investig..

[54] Mannini L., Lamaze F.C., Cucco F., Amato C., Quarantotti V., Rizzo I.M., Krantz I.D., Bilodeau S., Musio A. (2015). Mutant cohesin affects RNA polymerase II regulation in Cornelia de Lange syndrome. Sci. Rep..

[55] Musio A., Selicorni A., Focarelli M.L., Gervasini C., Milani D., Russo S., Vezzoni P., Larizza L. (2006). X-linked Cornelia de Lange syndrome owing to SMC1L1 mutations. Nat. Genet..

[56] Huisman S., Mulder P.A., Redeker E., Bader I., Bisgaard A.-M., Brooks A., Cereda A., Cinca C., Clark D., Cormier-Daire V. (2017). Phenotypes and genotypes in individuals with SMC1A variants. Am. J. Med. Genet. Part A.

[57] Krantz I.D., McCallum J., DeScipio C., Kaur M., Gillis L.A., Yaeger D., Jukofsky L., Wasserman N., Bottani A., Morris C.A. (2004). Cornelia de Lange syndrome is caused by mutations in NIPBL, the human homolog of Drosophila melanogaster Nipped-B. Nat. Genet..

[58] Selicorni A., Russo S., Gervasini C., Castronovo P., Milani D., Cavalleri F., Bentivegna A., Masciadri M., Domi A., Divizia M. (2007). Clinical score of 62 Italian patients with Cornelia de Lange syndrome and correlations with the presence and type of NIPBL mutation. Clin. Genet..

[59] Nizon M., Henry M., Michot C., Baumann C., Bazin A., Bessières B., Blesson S., Cordier-Alex M.-P., David A., Delahaye-Duriez A. (2016). A series of 38 novel germline and somatic mutations of NIPBL in Cornelia de Lange syndrome. Clin. Genet..

[60] Symonds J.D., Joss S., Metcalfe K.A., Somarathi S., Cruden J., Devlin A.M., Donaldson A., DiDonato N., Fitzpatrick D., Kaiser F.J. (2017). Heterozygous truncation mutations of the SMC1A gene cause a severe early onset epilepsy with cluster seizures in females: Detailed phenotyping of 10 new cases. Epilepsia.

[61] Deardorff M.A., Kaur M., Yaeger D., Rampuria A., Korolev S., Pie J., Gil-Rodríguez C., Arnedo M., Loeys B., Kline A.D. (2007). Mutations in Cohesin Complex Members SMC3 and SMC1A Cause a Mild Variant of Cornelia de Lange Syndrome with Predominant Mental Retardation. Am. J. Hum. Genet..

[62] Gil-Rodríguez M.C., Deardorff M.A., Ansari M., Tan C.A., Parenti I., Baquero-Montoya C., Ousager L.B., Puisac B., Hernández-Marcos M., Teresa-Rodrigo M.E. (2015). De Novo Heterozygous Mutations in SMC3 Cause a Range of Cornelia de Lange Syndrome-Overlapping Phenotypes. Hum. Mutat..

[63] De Falco A., De Brasi D., Della Monica M., Cesario C., Petrocchi S., Novelli A., D’alterio G., Iolascon A., Capasso M., Piscopo C. (2023). A Novel Variant in RAD21 in Cornelia De Lange Syndrome Type 4: Case Report and Bioinformatic Analysis. Genes.

[64] Kaiser F.J., Ansari M., Braunholz D., Decroos C., Wilde J.J., Fincher C.T., Kaur M., Bando M., Amor D.J., Atwal P.S. (2014). Loss-of-function HDAC8 mutations cause a phenotypic spectrum of Cornelia de Lange syndrome-like features, ocular hypertelorism, large fontanelle and X-linked inheritance. Hum. Mol. Genet..

[65] Parenti I., Gervasini C., Pozojevic J., Wendt K., Watrin E., Azzollini J., Braunholz D., Buiting K., Cereda A., Engels H. (2016). Expanding the clinical spectrum of the ‘*HDAC8*-phenotype’—Implications for molecular diagnostics, counseling and risk prediction. Clin. Genet..

[66] Whitehead M.T., Nagaraj U.D., Pearl P.L. (2015). Neuroimaging features of Cornelia de Lange syndrome. Pediatr. Radiol..

[67] Parenti I., Mallozzi M.B., Hüning I., Gervasini C., Kuechler A., Agolini E., Albrecht B., Baquero-Montoya C., Bohring A., Bramswig N.C. (2021). ANKRD11 variants: KBG syndrome and beyond. Clin. Genet..

[68] Parenti I., Diab F., Gil S.R., Mulugeta E., Casa V., Berutti R., Brouwer R.W., Dupé V., Eckhold J., Graf E. (2020). MAU2 and NIPBL Variants Impair the Heterodimerization of the Cohesin Loader Subunits and Cause Cornelia de Lange Syndrome. Cell Rep..

[69] Peng Y., Zhu Y., Wu L., Deng F. (2024). Clinical study and genetic analysis of Cornelia de Lange syndrome caused by a novel MAU2 gene variant in a Chinese boy. Mol. Genet. Genom. Med..

[70] Huisman S.A., Redeker E.J.W., Maas S.M., Mannens M.M., Hennekam R.C.M. (2013). High rate of mosaicism in individuals with Cornelia de Lange syndrome. J. Med. Genet..

[71] Ansari M., Poke G., Ferry Q., Williamson K., Aldridge R., Meynert A.M., Bengani H., Chan C.Y., Kayserili H., Avci Ş. (2014). Genetic heterogeneity in Cornelia de Lange syndrome (CdLS) and CdLS-like phenotypes with observed and predicted levels of mosaicism. J. Med. Genet..

[72] Pozojevic J., Parenti I., Graul-Neumann L., Ruiz Gil S., Watrin E., Wendt K.S., Werner R., Strom T.M., Gillessen-Kaesbach G., Kaiser F.J. (2018). Novel mosaic variants in two patients with Cornelia de Lange syndrome. Eur. J. Med. Genet..

[73] Latorre-Pellicer A., Gil-Salvador M., Parenti I., Lucia-Campos C., Trujillano L., Marcos-Alcalde I., Arnedo M., Ascaso Á., Ayerza-Casas A., Antoñanzas-Pérez R. (2021). Clinical relevance of postzygotic mosaicism in Cornelia de Lange syndrome and purifying selection of NIPBL variants in blood. Sci. Rep..

[74] Slavin T.P., Lazebnik N., Clark D.M., Vengoechea J., Cohen L., Kaur M., Konczal L., Crowe C.A., Corteville J.E., Nowaczyk M.J. (2012). Germline mosaicism in Cornelia de Lange syndrome. Am. J. Med. Genet. Part A.

[75] Krawczynska N., Wierzba J., Wasag B. (2019). Genetic Mosaicism in a Group of Patients With Cornelia de Lange Syndrome. Front. Pediatr..

[76] Mills J.A., Herrera P.S., Kaur M., Leo L., McEldrew D., Tintos-Hernandez J.A., Rajagopalan R., Gagne A., Zhang Z., Ortiz-Gonzalez X.R. (2018). NIPBL+/− haploinsufficiency reveals a constellation of transcriptome disruptions in the pluripotent and cardiac states. Sci. Rep..

[77] Sadikovic B., Levy M.A., Aref-Eshghi E. (2020). Functional annotation of genomic variation: DNA methylation episignatures in neurodevelopmental Mendelian disorders. Hum. Mol. Genet..

[78] Turinsky A.L., Choufani S., Lu K., Liu D., Mashouri P., Min D., Weksberg R., Brudno M. (2020). EpigenCentral: Portal for DNA methylation data analysis and classification in rare diseases. Hum. Mutat..

[79] Sadikovic B., Levy M.A., Kerkhof J., Aref-Eshghi E., Schenkel L., Stuart A., McConkey H., Henneman P., Venema A., Schwartz C.E. (2021). Clinical epigenomics: Genome-wide DNA methylation analysis for the diagnosis of Mendelian disorders. Genet. Med..

[80] Cerrato F., Sparago A., Ariani F., Brugnoletti F., Calzari L., Coppedè F., De Luca A., Gervasini C., Giardina E., Gurrieri F. (2020). DNA Methylation in the Diagnosis of Monogenic Diseases. Genes.

[81] Noh K.-M., Allis C.D., Li H. (2016). Reading between the Lines: “ADD”-ing Histone and DNA Methylation Marks toward a New Epigenetic “Sum”. ACS Chem. Biol..

[82] Haghshenas S., Bhai P., Aref-Eshghi E., Sadikovic B. (2020). Diagnostic Utility of Genome-Wide DNA Methylation Analysis in Mendelian Neurodevelopmental Disorders. Int. J. Mol. Sci..

[83] Sadikovic B. https://episign.lhsc.on.ca/can.html.

[84] Kerkhof J., Squeo G.M., McConkey H., Levy M.A., Piemontese M.R., Castori M., Accadia M., Biamino E., Della Monica M., Di Giacomo M.C. (2022). DNA methylation episignature testing improves molecular diagnosis of Mendelian chromatinopathies. Genet. Med..

[85] Avagliano L., Bulfamante G.P., Massa V. (2017). Cornelia de Lange syndrome: To diagnose or not to diagnose in utero?. Birth Defects Res..

[86] Panaitescu A.M., Duta S., Gica N., Botezatu R., Nedelea F., Peltecu G., Veduta A. (2021). A Broader Perspective on the Prenatal Diagnosis of Cornelia de Lange Syndrome: Review of the Literature and Case Presentation. Diagnostics.

[87] Hague J., Twiss P., Mead Z., Park S.-M. (2019). Clinical Diagnosis of Classical Cornelia de Lange Syndrome Made From Postmortem Examination of Second Trimester Fetus With Novel NIPBL Pathogenic Variant. Pediatr. Dev. Pathol..

[88] Parenti I., Teresa-Rodrigo M.E., Pozojevic J., Ruiz Gil S., Bader I., Braunholz D., Bramswig N.C., Gervasini C., Larizza L., Pfeiffer L. (2017). Mutations in chromatin regulators functionally link Cornelia de Lange syndrome and clinically overlapping phenotypes. Hum. Genet..

[89] Vasileiou G., Vergarajauregui S., Endele S., Popp B., Büttner C., Ekici A.B., Gerard M., Bramswig N.C., Albrecht B., Clayton-Smith J. (2018). Mutations in the BAF-Complex Subunit DPF2 Are Associated with Coffin-Siris Syndrome. Am. J. Hum. Genet..

[90] Conti B., Rinaldi B., Rimoldi M., Villa R., Iascone M., Gangi S., Porro M., Ajmone P.F., Colli A.M., Mosca F. (2023). Chung–Jansen syndrome can mimic Cornelia de Lange syndrome: Another player among chromatinopathies?. Am. J. Med. Genet. Part A.

[91] Xu B., Sowa N., Cardenas M.E., Gerton J.L. (2015). l-leucine partially rescues translational and developmental defects associated with zebrafish models of Cornelia de Lange syndrome. Hum. Mol. Genet..

[92] Pistocchi A., Fazio G., Cereda A., Ferrari L., Bettini L.R., Messina G., Cotelli F., Biondi A., Selicorni A., Massa V. (2013). Cornelia de Lange Syndrome: NIPBL haploinsufficiency downregulates canonical Wnt pathway in zebrafish embryos and patients fibroblasts. Cell Death Dis..

[93] Huang J., Nguyen-McCarty M., Hexner E.O., Danet-Desnoyers G., Klein P.S. (2012). Maintenance of hematopoietic stem cells through regulation of Wnt and mTOR pathways. Nat. Med..

[94] Avagliano L., Grazioli P., Mariani M., Bulfamante G.P., Selicorni A., Massa V. (2017). Integrating molecular and structural findings: Wnt as a possible actor in shaping cognitive impairment in Cornelia de Lange syndrome. Orphanet J. Rare Dis..

[95] Grazioli P., Parodi C., Mariani M., Bottai D., Di Fede E., Zulueta A., Avagliano L., Cereda A., Tenconi R., Wierzba J. (2021). Lithium as a possible therapeutic strategy for Cornelia de Lange syndrome. Cell Death Discov..

[96] de Graaf M., Kant S.G., Wit J.M., Redeker E.J.W., Santen G.W.E., Verkerk A.J.M.H., Uitterlinden A.G., Losekoot M., Oostdijk W. (2017). Successful Growth Hormone Therapy in Cornelia de Lange Syndrome. J. Clin. Res. Pediatr. Endocrinol..

[97] Cukrov D., Newman T.A.C., Leask M., Leeke B., Sarogni P., Patimo A., Kline A.D., Krantz I.D., Horsfield J.A., Musio A. (2018). Antioxidant treatment ameliorates phenotypic features of SMC1A-mutated Cornelia de Lange syndrome in vitro and in vivo. Hum. Mol. Genet..

[98] NCT04381897. NCT04381897.

[99] NCT05829668. NCT05829668.

[100] Ganesan A., Arimondo P.B., Rots M.G., Jeronimo C., Berdasco M. (2019). The timeline of epigenetic drug discovery: From reality to dreams. Clin. Epigenetics.

[101] Bondarev A.D., Attwood M.M., Jonsson J., Chubarev V.N., Tarasov V.V., Schiöth H.B. (2021). Recent developments of HDAC inhibitors: Emerging indications and novel molecules. Br. J. Clin. Pharmacol..

[102] Nuñez J.K., Chen J., Pommier G.C., Cogan J.Z., Replogle J.M., Adriaens C., Ramadoss G.N., Shi Q., Hung K.L., Samelson A.J. (2021). Genome-wide programmable transcriptional memory by CRISPR-based epigenome editing. Cell.

[103] Awamleh Z., Goodman S., Choufani S., Weksberg R. (2023). DNA methylation signatures for chromatinopathies: Current challenges and future applications. Hum. Genet..

